# Synchronous Hepatocellular Carcinoma and Cholangiocarcinoma in a Patient Transplanted for Cryptogenic Cirrhosis

**Published:** 2014-08-01

**Authors:** B. Geramizadeh, R. Gity, A. Bahraini, S. A. Malek-Hosseini

**Affiliations:** 1 Transplant Research Center; 2 Department of Pathology; 3 Department of Surgery, Shiraz University of Medical Sciences, Shiraz, Iran

## Abstract

Synchronous development of hepatocellular carcinoma (HCC) and cholangiocarcinoma (CC) in a liver is an extremely rare event. Very few cases have so far been reported in the literature with such condition. Herein, we report on the first case of incidental diagnosis of synchronous HCC and CC in a patient with cryptogenic cirrhosis who underwent liver transplantation. The diagnosis was made after careful examination of the explanted liver.

## INTRODUCTION

The most common primary cancer of the liver is hepatocellular carcinoma (HCC), which accounts for more than 80% of the primary liver malignancies. The second common primary liver cancer is cholangiocarcinoma (CC) [1]. There are reports of cancers with combined features of HCC and CC in a single tumor [2]. However, there are very few cases of synchronous occurrence of HCC and CC in a same liver in separate nodules [3].

Herein, we report on an extremely rare case of synchronous HCC and CC in two separate nodules of a cirrhotic liver. Both nodules were very small and discovered only after careful examination of the explanted liver. To the best of our knowledge, less than 35 such cases have so far been reported in the English literature.

## CASE REPORT

A 59-year-old man was referred to our center with the chief complaint of long-standing chills and fever. The patient has been a heavy smoker and been completely healthy prior to admission. During the last year, he experienced intermittent chills and fever, however he had not been visited by a physician. At hospitalization, his laboratory findings included an AST of 85 IU/L, ALT of 34 IU/L, alkaline phosphatase of 187 IU/L, BUN of 16 mg/dL, serum Cr of 8 mg/dL, WBC of 6300/µL, Hb of 10.7 g/dL, Plt count of 77,000/µL, PT of 18 sec, and PTT of 46 sec.

The anti-HCV and anti-HBV antibodies were negative. All tumor markers checked, including AFP and CA 19-9, were within normal limits.

Abdominal sonography showed a small liver with heterogeneous echo pattern. CT showed liver cirrhosis with mild splenomegaly but without any obvious mass lesions in the liver.

After liver biopsy that confirmed the diagnosis of cryptogenic cirrhosis, he was put in liver transplantation waiting list during which period, he experienced several episodes of decreased level of consciousness. 

**Figure 1:**
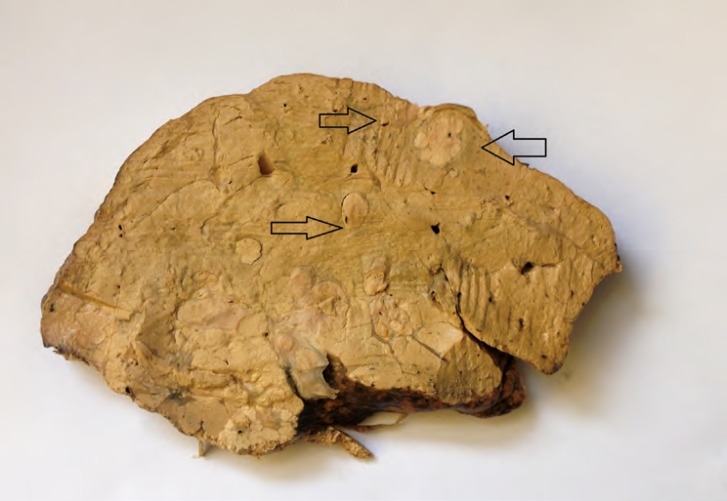
Gross view of the liver shows the three nodules. The upper right nodule and the lower one are HCC; the upper left one is CC.

After liver transplantation, the explanted liver was sent for pathological examination. Thorough examination of the formalin-fixed explanted cirrhotic liver showed three discrete ill-defined lesions in the upper and lower parts of the right lobe, each measuring about 1.5 cm in diameter. The upper two masses though were very close to each other had not connection at all (Fig 1).

**Figure 2:**
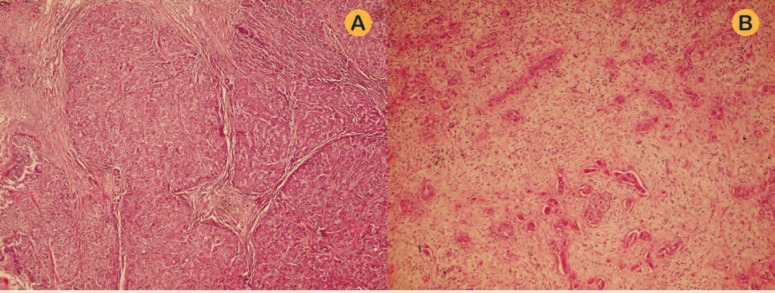
A) Nodule of HCC, B) nodule of CC (H&E ×100)

**Figure 3:**
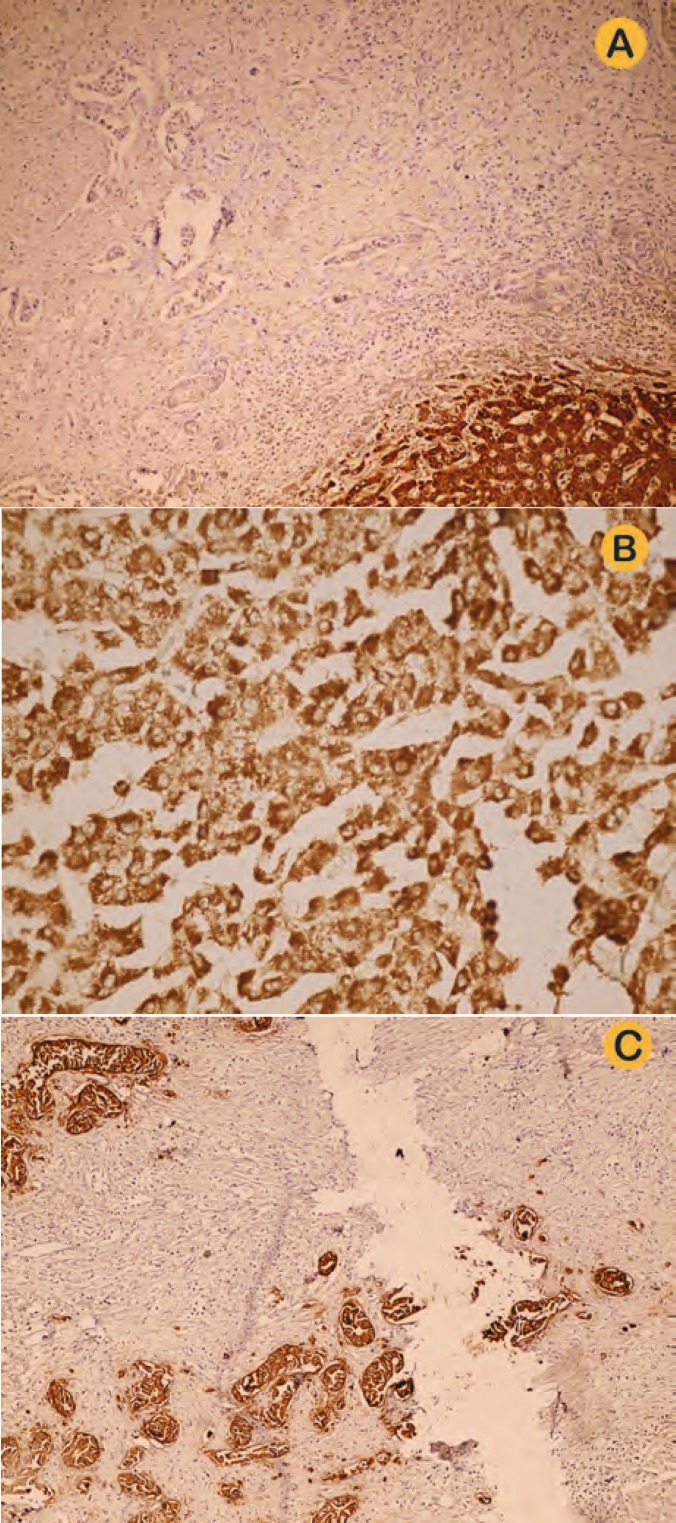
A) IHC for HepPar-1 in CC nodule; B) IHC for HepPar-1 in HCC nodule; and C) IHC for MOC-31 in CC nodule

Microscopic examination of the nodules showed three completely separated tumors—two typical for HCC and the other one typical for CC (Fig 2A, B). Immunohistochemical study showed positive HepPar-1 and paracanalicular CEA in one nodule and negative HepPar-1 and positive cytoplasmic CEA and CK7 and CK19 in the other nodule (Fig 3A, B, C).

All the resected margins including vascular and biliary structures of hilum were free of any tumor involvement. With the diagnosis of synchronous HCC and CC, the patient was followed. Now after a year, he is still doing well and under treatment for ordinary transplanted liver with no episode of significant rejection.

## DISCUSSION

Synchronous tumors in a same organ is an extremely rare occurrence. In the liver, although CC and HCC are the two main primary liver tumors, synchronous development of HCC and CC in the same liver is very rare so that less than 35 cases have so far been reported in the literature. Most of the previous cases were reported from Korea and Japan [1]. 

Two theories have been proposed for such a co-occurrence. One is the appearance of amphi-potential progenitor cells with differentiation and proliferation in the tumor and the other theory is the malignant transformation of the cells which have already differentiated into both cholangiocytes and hepatocytes [2].

Most of the previously reported cases were on the base of cirrhosis secondary to hepatitis C, however, rare cases with hepatitis B-related cirrhosis [3], and also cases with no underlying cirrhosis have been reported [4]. Therefore, it seems that HCV is a risk factor not only for HCC but also for CC [5].

All of the previously reported cases, and also our patient, aged more than 50 years; most of them were male [1]. Most of the previous cases were diagnosed by imaging before surgery [6]; to the best of our knowledge, none of the reported cases were incidentally found after liver transplant. 

There were cases who have been operated with the impression of double nodules of HCC, but after pathological examination, CC was found [7]. There is also report of the co-occurrence of HCC and CC in two separate dysplastic nodules in a patient with HCV-related cirrhosis [8]. Another risk factor for the development of this rare co-occurrence is von-Meyen-Berg complex [9].

The best treatment for synchronous HCC and CC is surgery; in particular, when both tumors are resectable, there are good results after surgery [10].

As a conclusion, co-occurrence of HCC and CC in a same liver in separate nodules should be considered in the hepatectomy specimens after liver transplantation. Therefore, careful examination and sectioning of all of the nodules should be performed.
